# Case of Supra-pubic Lithotomy in a Woman—An Exceptional Case

**Published:** 1884-12

**Authors:** Lawson Tait

**Affiliations:** Surgeon to the Birmingham and Midland Hospital for Women; Consulting Surgeon to the West Bromwich Hospital


### CASE OF SUPRA-PUBIC LITHOTOMY IN A WOMAN—AN EXCEPTIONAL CASE.

By LAWSON TAIT, F R. C. S.,

Surgeon to the Birmingham and Midland Hospital for Women ; Consulting Surgeon to the West Bromwich Hospital.

J. M., æt. 24, was confined in September, 1881, being attended by an irregular practitioner, after a labor of about ninety hours. She came under my care in January, 1882, at the Hospital for Women, as an out-patient, and I found that the whole of the posterior wall of the bladder had been destroyed, leaving only a fragment of the trigone and the urethia. A large amount of the posterior wall of the vagina had also been destroyed. I admitted her, and by a series of plastic operations, seven in number, and consisting chiefly of bringing flaps from the posterior and lateral parts of the vagina, I managed to build up a new posterior wall for her bladder, and to fasten it to the stump of the trigone. I had to turn the remains of the cervix uteri into the bladder, and when the recovery was complete there was very little vagina, and menstruation occurred into the bladder. That viscus, however, in the course of a few months became large enough to contain nearly eight ounces of urine, and it was completely under her control as long as its contents did not exceed that amount. Her existence, therefore, became one of comparative comfort, and remained so until she had to accompany her husband, a soldier, to Ireland. She came back to me in March of this year with symptoms of stone in the bladder, and I easily found and crushed a large stone with great but not with complete relief. After several soundings I found another stone (possibly a fragment of the first) in the posterior and upper part of the bladder. I could only touch it occasionally in certain positions, and I could not lay hold of it with the lithotrite, though I tried many times. I became certain, therefore, that it was lodged in a pocket made, in all probability, by the puckering of my artificial work in closing her bladder. The question came, how was I to get at it? Vaginal lithotomy was out of the question, as I must of necessity undo all my old success. Dilating the urethra I do not like, and if I did it in this case there was the possibility that even then I might fail to get at the stone in its pocket. I therefore determined to open the bladder from above. I had never performed supra-pubic lithotomy, and I had never heard of its being done in a woman. I have opened the bladder several times unwittingly in removing uterine tumors, fortunately never with any ill result. I have seen supra-pubic lithotomy performed only twice; in both cases the peritoneum was opened, and both patients died. I am not afraid of opening the peritoneum; still I was anxious to avoid doing so, and therefore I kept very close to the pubis, and an assistant pressed the fundus of the bladder up with a sound passed through the urethra. With all my precautions, I did open the peritoneum, and can easily understand how this method of removing stones from the bladder has never become a popular one. I found the stone in a pocket on the left side of the cervix, in such a position that I could not have removed it, at least not easily, if I had passed my finger from the urethra. I closed the wound in the peritoneum very carefully, and passed a drainage tube through the urethra and out at the wound in the base of the bladder. The patient has made an excellent recovery; the supra-pubic wound is now nearly healed, and she passes water without any discomfort. The stone was, as usual, a very soft phosphatic concretion, and was adherent to the wall of the sac in which it was situated.

In such a case as this the tendency to the formation of stone is very great, and in many of my bad cases of vesico-vaginal fistula I have had to do lithotrity at intervals after I had closed the aperture. Lithotrity in women is such a perfectly easy operation that until now no difficulty has arisen; but in this exceptional case I think I adopted the only possible course.—*Medical Press.*

